# Changes in multimodality functional imaging parameters early during chemoradiation predict treatment response in patients with locally advanced head and neck cancer

**DOI:** 10.1007/s00259-017-3890-2

**Published:** 2017-11-21

**Authors:** Kee H. Wong, Rafal Panek, Alex Dunlop, Dualta Mcquaid, Angela Riddell, Liam C. Welsh, Iain Murray, Dow-Mu Koh, Martin O. Leach, Shreerang A. Bhide, Christopher M. Nutting, Wim J. Oyen, Kevin J. Harrington, Kate L. Newbold

**Affiliations:** 1 Head and Neck Unit, The Royal Marsden NHS Foundation Trust, Downs Road, Sutton, SM2 5PT UK; 2 Radiotherapy and Imaging Division, The Institute of Cancer Research, London, UK; 3 Clinical Radiology, The Royal Marsden NHS Foundation Trust, Sutton, UK; 4 Nuclear Medicine, The Royal Marsden NHS Foundation Trust, Sutton, UK; 5 CRUK Cancer Imaging Centre, The Institute of Cancer Research and the Royal Marsden NHS Foundation Trust, London, UK

**Keywords:** Head and neck squamous cell carcinoma, MRI, PET/CT, Chemoradiation, Biomarker

## Abstract

**Objective:**

To assess the optimal timing and predictive value of early intra-treatment changes in multimodality functional and molecular imaging (FMI) parameters as biomarkers for clinical remission in patients receiving chemoradiation for head and neck squamous cell carcinoma (HNSCC).

**Methods:**

Thirty-five patients with stage III-IVb (AJCC 7th edition) HNSCC prospectively underwent ^18^F–FDG-PET/CT, and diffusion-weighted (DW), dynamic contrast-enhanced (DCE) and susceptibility-weighted MRI at baseline, week 1 and week 2 of chemoradiation. Patients with evidence of persistent or recurrent disease during follow-up were classed as non-responders. Changes in FMI parameters at week 1 and week 2 were compared between responders and non-responders with the Mann–Whitney U test. The significance threshold was set at a *p* value of <0.05.

**Results:**

There were 27 responders and 8 non-responders. Responders showed a greater reduction in PET-derived tumor total lesion glycolysis (TLG_40%_; *p* = 0.007) and maximum standardized uptake value (SUV_max_; *p* = 0.034) after week 1 than non-responders but these differences were absent by week 2. In contrast, it was not until week 2 that MRI-derived parameters were able to discriminate between the two groups: larger fractional increases in primary tumor apparent diffusion coefficient (ADC; *p* < 0.001), volume transfer constant (K^trans^; *p* = 0.012) and interstitial space volume fraction (V_e_; *p* = 0.047) were observed in responders versus non-responders. ADC was the most powerful predictor (∆ >17%, AUC 0.937).

**Conclusion:**

Early intra-treatment changes in FDG-PET, DW and DCE MRI-derived parameters are predictive of ultimate response to chemoradiation in HNSCC. However, the optimal timing for assessment with FDG-PET parameters (week 1) differed from MRI parameters (week 2). This highlighted the importance of scanning time points for the design of FMI risk-stratified interventional studies.

**Electronic supplementary material:**

The online version of this article (10.1007/s00259-017-3890-2) contains supplementary material, which is available to authorized users.

## Introduction

Radical chemoradiation (CRT) is widely accepted as the standard of care for organ-sparing treatment of locally advanced head and neck squamous cell carcinoma (HNSCC). However, it is now evident that locally advanced HNSCC represents a disease spectrum rather than a single entity, with variable response to standard CRT. Clinical variables such as tumor, node and metastases (TNM) staging and smoking history are prognostically robust but predictively deficient. Reliable prediction of outcome early during CRT is, therefore, highly desirable to avoid continuation of ineffective treatment and guide adaptation of therapy based on response.

Functional and molecular imaging (FMI) can characterize tumor phenotypes by providing quantitative parameters with radiobiological relevance. ^18^F–FDG-PET/CT and MRI both have the benefit of being non-invasive, allowing serial measurements during radiotherapy. Several correlation studies in HNSCC have demonstrated varied, but complementary, biological information from different FMI parameters [1, 2]. To date, most FMI biomarker studies in HNSCC have concentrated only on pre-treatment time points. Indeed, there remains a paucity of prospective data to define the optimal time point for early intra-treatment assessment in patients receiving CRT.

Here, we report the results of serial ^18^F–FDG-PET/CT, and diffusion-weighted (DW), dynamic contrast-enhanced (DCE) and susceptibility-weighted (SW) MRI following the first and second week of CRT in patients with HNSCC. The primary objective of this analysis is to identify the optimal timing and predictive value of early intra-treatment changes in FMI parameters for ultimate response to CRT.

## Materials and methods

### Study design

Patients with previously untreated histologically proven HNSCC (AJCC 7th edition stage III-IVb) and WHO performance status 0–2 planned for CRT, were eligible for the study. Forty patients were recruited at our institution between April 2014 and August 2016. This study received approvals from the institutional review board (CCR3926) and research ethical committee (13/LO/0067). All patients provided written consent.

Patients were treated with 6 weeks of radiotherapy with concomitant chemotherapy [cisplatin (100 mg/m^2^) or carboplatin (AUC5) days 1 and 29]. Macroscopic and microscopic disease received 65 Gy and 54 Gy in 30 fractions, respectively, using intensity-modulated radiotherapy with a simultaneous integrated boost technique.

Patients prospectively underwent ^18^F–FDG-PET/CT, DW, SW and DCE MRI at baseline, week 1 (after the 4th or 5th fraction) and week 2 (after the 9th or 10th fraction) during treatment. Response was assessed at 3 months following completion of CRT with MRI, ^18^F–FDG-PET/CT and clinical examination including nasendoscopy. Patients with evidence of residual disease at 3 months were discussed in the multidisciplinary meeting for feasibility of salvage surgery. Patients were followed up for a total of 2 years within this study.

### PET/CT image acquisition

^18^F–FDG-PET/CT studies were acquired using Phillips Gemini (London) and Siemens mCT (Sutton) PET/CT scanners. Patients were fasted for 6 h before the study. The ^18^F–FDG dose was determined according to EARL guidelines [3] and was administered intravenously if the blood sugar level was <10 mmol/L. Subsequently, patients rested for 60 min. Patients were positioned on a flat-top couch in the radiotherapy treatment position, using a headrest and 5-point thermoplastic shell. Unenhanced, low-dose CT was performed from the vertex to carina for purposes of attenuation correction and image fusion for anatomical localisation (approximate mAs 50/slice). FDG emission data were acquired from the vertex to carina (3 min/bed; average 2-bed acquisition).

### MRI image acquisition

All MRI scans were acquired on a 1.5-T scanner (MAGNETOM Aera, Siemens Healthcare, Erlangen, Germany). Patients were set up on a flat-top MRI couch in the radiotherapy treatment position, using a headrest and 5-point thermoplastic shell. A large flex and spine coils were used. For all images, a 200 × 200-mm field of view (FOV), 2-mm isotropic voxel size and 80-mm cranio-caudal coverage was used. Anatomical T_2_-weighted [echo time/repetition time (TE/TR): 82/11000 ms] and T_1_-weighted (TE/TR: 13/794 ms) were acquired first to aid functional MRI planning. DW-MRI sequences [SE-EPI DWI, TE/TR: 61/13400, b values: 50, 400 and 800 s/mm^2^, monopolar diffusion gradients, number of signal averages (NSA) = 5, bandwidth (BW) = 1000 Hz, matrix 96] were then acquired.

T_2_* (R_2_* = 1/T_2_*) was measured using a 2D gradient echo sequence with eight echo times [flip angle (FA) = 24, TE 4.76 to 38.08 ms in increments of 4.76 ms,

TR = 1990 ms, BW = 400 Hz]. The DCE protocol included a trans-axial 3D spoiled fast gradient echo sequence with DIXON fat and water signal separation and TWIST under-sampling [TE = 2.4 and 4.8 ms, TR = 7.2 ms, BW = 450 Hz, TWIST A&B = 33%, CAIPIRIHNA (*R* = 4)]. A series of 12 proton density-weighted images (FA = 4°) was initially acquired, followed by 100 T_1_-weighted acquisitions (FA = 24°) obtained sequentially with 2-s temporal resolution. Gadolinium (Gd) contrast agent was administered intravenously at the start of the 11th dynamic scan as a bolus through a peripherally placed cannula using an automatic injector (0.2 mL/kg body mass, 2-mL/s injection rate; Dotarem, Guerbet, France) and followed by a saline flush (20 mL at 2 mL/s). A full blood count was taken prior to each MRI scan to determine blood hematocrit levels.

### Image analysis

Both PET and MRI data were analyzed using RayStation (version 4.9.9, RaySearch Medical Laboratories, AB Stockholm, Sweden), a radiotherapy treatment planning system. Regions of interest (ROIs) encompassing the primary tumor (PT) or/and involved lymph nodes (LNs) were delineated on each imaging modality by a radiation oncologist (KW). These contours were verified by respective consultants in nuclear medicine (WO) and radiology (AR). LNs included in the analysis were either proven via cytology/histology results or deemed to be unequivocally involved based on both anatomical and functional imaging characteristics following consensus among investigators.

PET images reconstructed using ordered subset expectation maximization were used for analysis. A relative threshold of 40% of the maximum standardized uptake volume (SUV_max_) was used to generate the metabolic tumor volume (MTV_40%_). The baseline value served as the threshold for MTV on subsequent scans. PET parameters including SUVmax and total lesion glycolysis (TLG_40%_ = SUV_mean_ x MTV_40%_) [4], were recorded for all ROIs at each time point.

Anatomical contours for MRI were delineated on T_2_-weighted images with reference to T_1_-weighted images. For DW analysis, ROIs were defined on the T_2_-weighted low-b value images (b50) with reference to the co-registered anatomical contours, excluding regions of macroscopic necrosis and cystic change. All b values were used to calculate the apparent diffusion coefficient (ADC). Signal changes on multiple-gradient echo images were used to produce spatial R_2_* parametric maps. Signal intensity decay measured for increasing echo times was fitted on a voxel-by-voxel basis to a monoexponential model using a least-squares fit method. This was performed using in-house MatLab software (MathWorks, Natick, MA, USA). The DCE data were analyzed using the MRIW software package developed by The Institute of Cancer Research (ICR) [5]. The extended Kety model [6] and a population-based arterial input function [7] were used to derive a set of parameters, including the volume transfer constant between blood plasma and extracellular extravascular space (K^trans^), the total extracellular extravascular space volume fraction (V_e_) and the total blood plasma volume fraction (V_p_). For SW and DCE analysis, ROIs were defined on co-registered T_1_ post-Gd images.

Due to the skewed distribution of parameter values, the median was chosen as the statistical representation for each individual ROI. The fractional changes in FMI parameters from baseline [Δ = (x – baseline)/baseline] were calculated for each scanning time point. The corresponding radiotherapy planning CT and dosimetry were also imported to enable dosimetric analysis.

### Statistical analysis

The data were analyzed using SPSS statistical software (Version 24.0; IBM Corp, Armonk, NY, USA). The fractional changes in FMI parameters at week 1 and week 2 were compared between responders and non-responders using the Mann–Whitney U test. Categorical data were compared between the two groups using the Pearson Chi-squared test. The significance threshold was set at *p* < 0.05. Receiver operator characteristic (ROC) analysis was used to identify and determine the optimal threshold values for parameters with predictive value.

## Results

### Clinical characteristics

Following exclusion of 5 patients who failed to undergo intra-treatment scans, 35 patients were available for analysis. The main reason for non-attendance was treatment-induced acute side-effects. The average times from start of CRT to scanning time points for week 1 and week 2 were 6.4 ± 1.0 days and 13.3 ± 1.0 days, respectively. Patient and tumor characteristics are summarized in Table 1. All patients completed CRT within 42 days. One patient was switched from carboplatin to cetuximab due to suboptimal renal function. The median follow-up was 14 months (range 5–33).

**Table 1:** Summary of patient and tumor characteristics

Number of patients	35
Age (y), median (range)	61 (34–69)
Sex (%)	
Male	35 (100)
Female	0 (0)
Primary location (%)	
Oropharynx	29 (83)
Hypopharynx/larynx	6 (17)
HPV status (%)	
Positive	22 (63)
Negative	13 (37)
T classification (%)	
1–2	20 (57)
3–4	15 (43)
N classification (%)	
0–1	12 (34)
2–3	23 (66)
Concomitant chemotherapy (%)	
Cisplatin	15 (43)
Carboplatin	10 (28)
Cis/carbo*	9 (26)
Cetuximab	1 (3)

*Cisplatin on day 1 switched to carboplatin on day 29

### Treatment outcome

There were 27 responders and 8 non-responders: 5 non-responders had locoregional failure only, 2 had both locoregional and distant failure and 1 had distant metastases without any detectable disease above the clavicles. Review of the radiotherapy dosimetry in the non-responders with locoregional persistence/recurrence revealed that all failures were in-field, i.e. within clinical target volume receiving 65 Gy. Only one non-responder was deemed suitable for salvage surgery and underwent total laryngectomy with bilateral neck dissection. That patient remains disease-free to date. Four non-responders had died at the time of analysis (5–15 months post-CRT).

### Responders versus non-responders

In this cohort, treatment-induced changes in anatomical tumor volume in the first 2 weeks of CRT failed to discriminate responders from non-responders (Supplementary Table 1). Similar negative results were seen for clinical characteristics such as HPV status and T and N classification (*p* > 0.05, Supplementary Table 2).

Responders showed a greater reduction in tumor TLG_40%_ (*p* = 0.007) and SUV_max_ (*p* = 0.034) after week 1 of CRT than non-responders (Table 2). These differences between the two groups, however, disappeared by week 2. When the data were separately analyzed per PT and LNs, similar trends were observed but these failed to reach statistical significance.

**Table 2:** Comparison of PET parameters between responders and non-responders (*n* = 35)

FMI parameters	Responders (%)	Non-responders (%)	*p* value	ROC (AUC)
Combined (PT + LNs)				
TLG_40%_ (g)				
Baseline	112.4 ± 98.2	264.7 ± 361.0%	0.192	–
Δ post-wk 1	−56.3 ± 31.0	−18.0 ± 38.8	0.007*	0.825
Δ post-wk 2	−68.7 ± 20.9	−48.8 ± 33.3	0.131	–
SUV_max_				
Baseline	14.2 ± 5.2	16.6 ± 5.9	0.269	–
Δ post-wk 1	−27.8 ± 21.1	−8.1 ± 18.8	0.034*	0.764
Δ post-wk 2	−32.5 ± 23.0	−21.4 ± 19.8	0.158	–
Primary tumor (PT)				
TLG_40%_ (g)				
Baseline	87.8 ± 80.1	233.5 ± 291.2	0.104	–
Δ post-wk 1	−41.3 ± 23.5	−18.3 ± 37.7	0.069	–
Δ post-wk 2	−60.2 ± 17.1	−48.2 ± 34.9	0.310	–
SUV_max_				
Baseline	14.7 ± 4.7	16.4 ± 6.1	0.498	–
Δ post-wk 1	−20.6 ± 12.7	−4.9 ± 18.5	0.050	–
Δ post-wk 2	−27.7 ± 14.7	−20.5 ± 18.0	0.224	–
Lymph nodes (LNs)				
TLG_40%_ (g)				
Baseline	50.6 ± 57.8	70.9 ± 88.4	0.532	–
Δ post-wk 1	−56.5 ± 39.5	−16.6 ± 50.9	0.085	–
Δ post-wk 2	−65.6 ± 28.2	−52.9 ± 45.6	0.790	–
SUV_max_				
Baseline	10.9 ± 5.1	11.8 ± 5.2	0.859	–
Δ post-wk 1	−32.1 ± 24.1	−18.8 ± 16.6	0.391	–
Δ post-wk 2	−34.8 ± 28.1	−34.9 ± 12.2	0.533	–

*Statistically significant difference

**Fig. 1:**
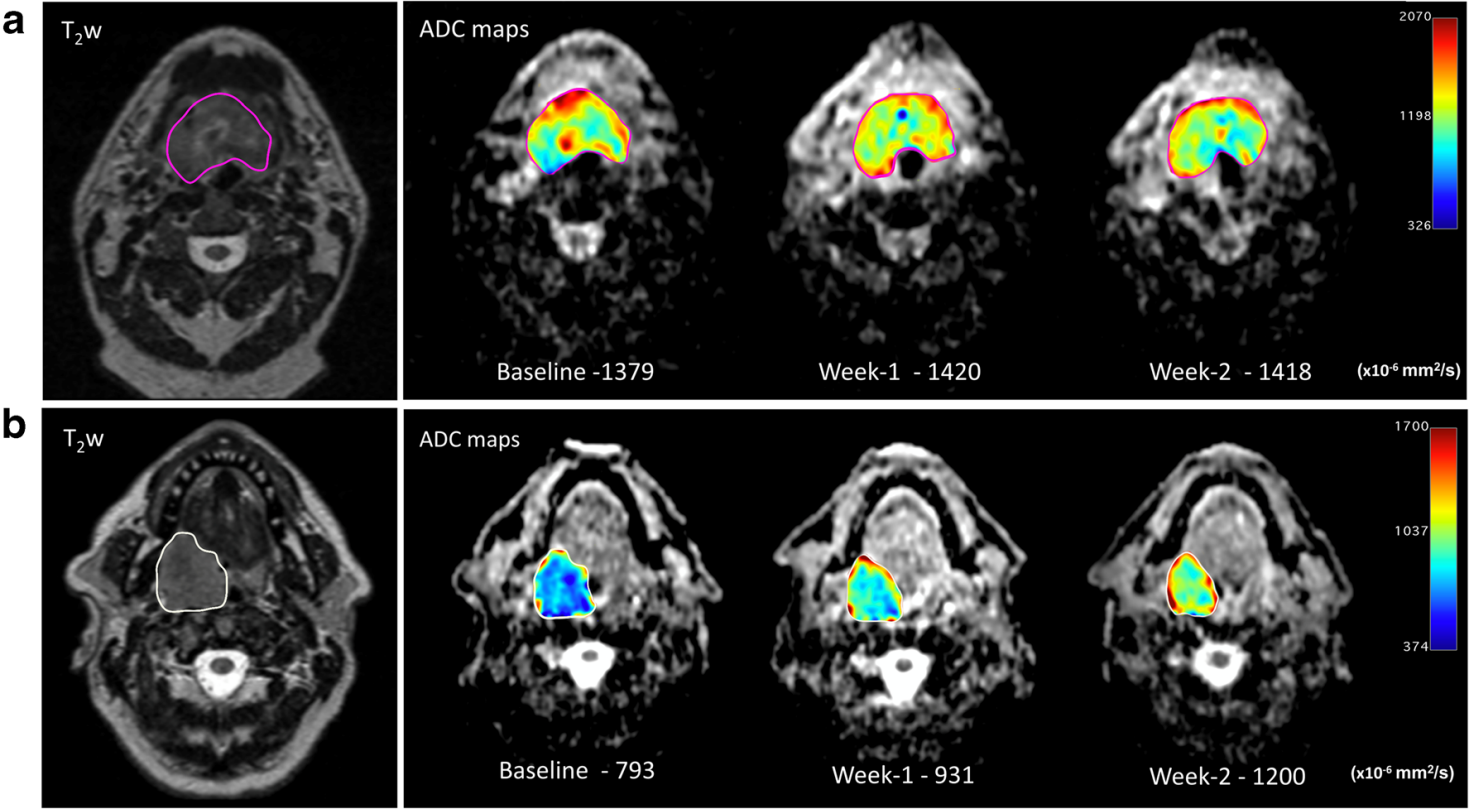
Serial ADC maps (tumor ROI displayed in jet color scale) in the first 2 weeks of CRT for **A** (non-responder) and **B** (responder). Patient B showed a large treatment-induced increase in PT ADC (+51.3% post-week 2) in contrast to patient A (+2.8% post-week 2)

**Fig. 2:**
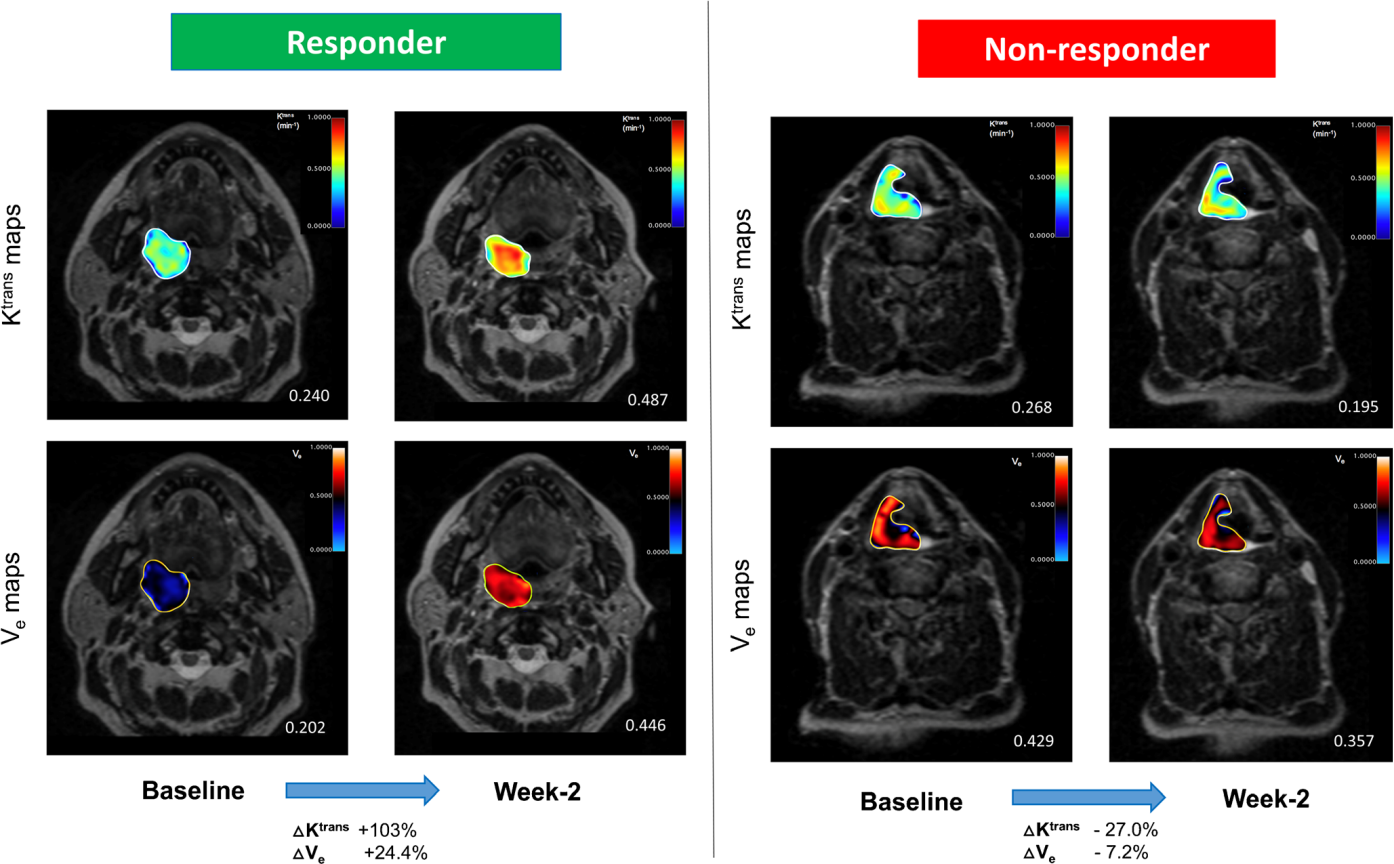
Overlay K^trans^ and V_e_ maps demonstrating the differences in longitudinal changes between responder and non-responder after week 2 of CRT. The responder showed a significantly larger increase in median K^trans^ and V_e_, in comparison to the non-responder

The fractional changes in functional MRI parameters during CRT are summarized in Table 3. Responders showed a larger fractional increase in PT ADC values at week 2 than non-responders (33.7 ± 15.1% versus 11.0 ± 9.0%, *p* < 0.001). Whilst there was a similar trend at week 1, it was not discriminative between responders and non-responders. An example of serial changes in PT ADC is illustrated in Fig. 1. In addition, significantly larger increases in PT K^trans^ (*p* = 0.012) and V_e_ (*p* = 0.047) at week 2 were observed in responders (Fig. 2). Similarly, the differences in these parameters between the two groups were absent at week 1.

**Table 3:** Comparison of MRI parameters between responders and non-responders (n = 35)

FMI parameters	Responders (%)	Non-responders (%)	*p* value	ROC (AUC)
Primary (27 ROIs)				
ADC (×10^−3^ mm^2^/s)				
Baseline	1.02 ± 0.19	1.22 ± 0.14	0.009*	0.829
Δ post-wk 1	18.9 ± 25.5	5.1 ± 33.7	0.274	–
Δ post-wk 2	33.7 ± 15.1	11.0 ± 9.0	0.000*	0.937
R_2_*(s^−1^)				
Baseline	34.3 ± 10.5	31.4 ± 10.0	0.685	–
Δ post-wk 1	0.7 ± 23.6	−5.3 ± 25.6	0.555	–
Δ post-wk 2	−5.3 ± 25.6	4.0 ± 25.4	0.791	–
K^trans^ (min^−1^)				
Baseline	0.202 ± 0.040	0.194 ± 0.049	0.735	–
Δ post-wk 1	47.3 ± 41.6	28.2 ± 27.3	0.331	–
Δ post-wk 2	115.4 ± 117.2	13.9 ± 51.2	0.012*	0.813
V_e_				
Baseline	0.256 ± 0.055	0.318 ± 0.055	0.003*	0.864
Δ post-wk 1	6.9 ± 5.9	8.1 ± 22.9	0.836	–
Δ post-wk 2	12.9 ± 9.8	0.1 ± 16.6	0.047*	0.774
V_p_ (×10^−3^)				
Baseline	8.5 ± 7.4	2.7 ± 5.6	0.072	–
Δ post-wk 1	−0.52 ± 0.73	−0.03 ± 0.86	0.219	–
Δ post-wk 2	−0.53 ± 0.75	−0.16 ± 2.06	0.288	–
Lymph nodes (36 ROIs)				
ADC (×10^−3^ mm^2^/s)				
Baseline	1.059 ± 0.211	1.086 ± 0.091	0.456	–
Δ post-wk 1	13.6 ± 12.2	18.0 ± 7.4	0.239	–
Δ post-wk 2	33.4 ± 19.0	30.1 ± 4.4	0.900	–
R_2_*(s^−1^)				
Baseline	20.7 ± 4.7	21.1 ± 2.3	0.611	–
Δ post-wk 1	3.8 ± 18.4	4.5 ± 10.8	0.677	–
Δ post-wk 2	4.4 ± 23.0	7.4 ± 8.3	0.445	–
K^trans^ (min^−1^)				
Baseline	0.167 ± 0.044	0.204 ± 0.81	0.184	–
Δ post-wk 1	45.5 ± 42.5	64.6 ± 96.4	1.000	–
Δ post-wk 2	34.5 ± 59.7	−5.0 ± 31.3	0.102	–
V_e_				
Baseline	0.241 ± 0.079	0.339 ± 0.120	0.020*	0.783
Δ post-wk 1	6.8 ± 7.1	13.8 ± 14.7	0.244	–
Δ post-wk 2	11.9 ± 10.3	8.5 ± 4.3	0.694	–
V_p_ (×10^−3^)				
Baseline	4.5 ± 5.2	1.1 ± 2.6	0.229	–
Δ post-wk 1	−0.18 ± 0.50	−0.13 ± 0.28	0.944	–
Δ post-wk 2	−0.12 ± 0.67	−0.10 ± 0.30	0.813	–

*statistically significant difference

Conversely, the changes in R_2_* early during CRT appeared random with no apparent trend (Supplementary Fig. 1). Whilst there was a trend for a larger decrease in PT V_p_ in responders, this did not reach statistical significance. No significant intra-treatment changes were detected in LNs for all functional MRI parameters; however, the trends were consistent for those for PTs.

ROC analysis identified changes in PT ADC at week 2 as the most powerful predictor of response to CRT with AUC of 0.937. An increase in PT ADC >17% at week 2 has a sensitivity, specificity and accuracy of 100%, 86% and 96%, respectively, in predicting response following CRT. For earlier assessment at week 1, total TLG_40%_ was the parameter of choice with a reduction of >12%, giving a sensitivity and specificity of 93% and 83%, respectively, in predicting treatment response. Attempts to combine the strongest predictor, ADC, with other FMI parameters did not further improve its performance.

## Discussion

We evaluated early intra-treatment assessment using multimodality FMI parameters to predict response to CRT in patients with locally advanced HNSCC. The reason for investigating the chosen study time points and not later, i.e. >2 weeks, is to allow early identification of patients who are likely or unlikely to respond so that the window of opportunity to affect therapeutic outcome is not missed. Responders could be considered for treatment de-escalation, e.g. radiotherapy dose reduction or target volume adaptation, to reduce treatment-related morbidity [8, 9]. In contrast, non-responders should be considered for treatment intensification, e.g. radiotherapy dose escalation [10], hypoxia modification [11], novel radiosensitisers [12, 13] and/or ‘bail out’ surgery.

**Fig. 3:**
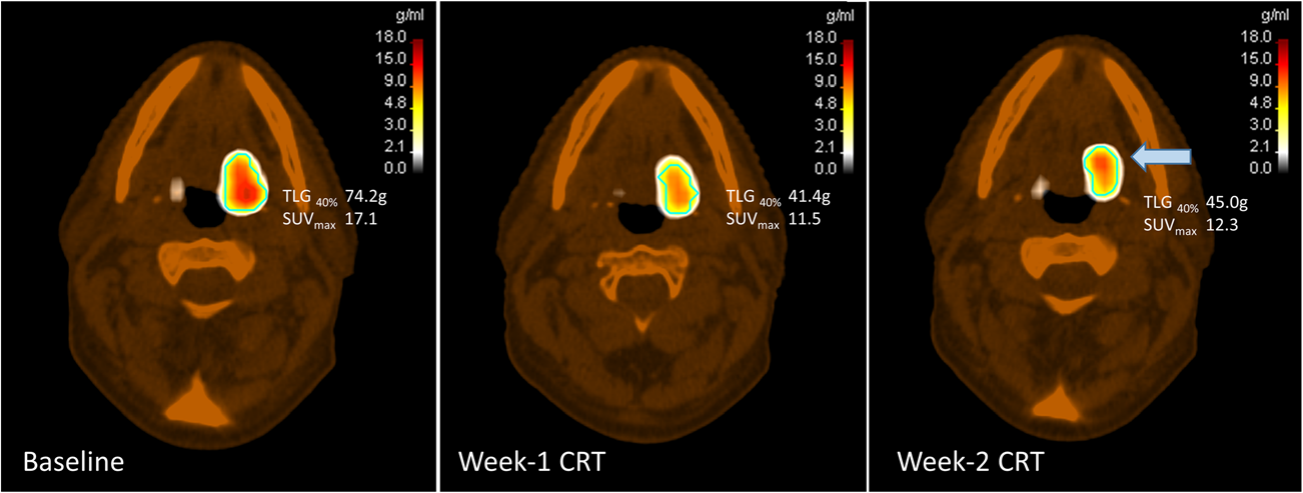
An example of a responder having a paradoxical increase in primary tumor SUV at week 2 (highlighted by the blue arrow, cyan contour = MTV_40%_) despite a marked initial decrease at week 1. This phenomenon was observed in a few other responders, which may be confounded by radiotherapy-induced peritumoral inflammation with cumulative fractions

As shown in previous studies [14, 15], treatment-induced changes in FMI parameters precede anatomical changes, allowing earlier risk stratification of patients. Our data demonstrated differing optimal times for early response assessment during CRT when FDG-PET and MRI parameters were used. Changes in tumor TLG_40%_ and SUV_max_ at week 1 were predictive of treatment outcome, but these signals, in fact, disappeared later by week 2. This was explained by the low tumor FDG uptake and lesser difference between the two groups by week 2. Another possible confounding factor is the influence of radiotherapy-induced peritumoral inflammation on FDG uptake with cumulative fractions, which may affect ROI segmentation (Fig. 3), but this phenomenon is typically observed during the latter part of radiotherapy [2, 16]. This raises uncertainties about the reliability of FDG-PET parameters in reflecting tumor response beyond the first week of CRT. It is also evident that combined, rather than isolated, analysis of PT and LN FDG-PET parameters provides a better overall representation of the tumor response. To the best of our knowledge, this is the first published data investigating the predictive value of early intra-treatment changes in FDG-PET parameters in such a setting.

In contrast to FDG-PET parameters, it was not until week 2 that treatment-induced changes in MRI parameters successfully discriminated responders from non-responders. Our data showed that a larger fractional increase in PT DW-derived ADC at week 2 (∆ > 17%) was highly predictive of favorable response to CRT. Overall, our results are consistent with previous DW MRI studies which cumulatively reported that an increase of tumor ADC (>14–24%) between week 1 and 4 of radiotherapy could predict treatment outcome [17–20]. Kim et al. reported intra-treatment assessment at week 1 to be predictive of response, but their scans were performed on average 12 days after the start of CRT [19], which would have been defined as week 2 in our study. Thus, it is reasonable to deduce that whilst highly desirable, earlier assessment with DW MRI (e.g. <7 days from start of CRT) is premature and of limited utility in HNSCC. Moreover, our study improves on previous studies due to its homogeneity: previous studies used MRI of different strengths (1.5 and 3 T) within the same study, had less standardized scanning time points (standard deviation of >3 days) or/and included patients with early disease (stage I-II) undergoing radiotherapy only.

We also found responders to have a larger fractional increase in PT DCE-derived K^trans^ and V_e_. Similarly, these were only evident by week 2. These observations are likely to reflect early cell degradation in responding tumors, resulting in expansion of interstitial space and increased vascular permeability. Unlike DW MRI, there is limited data on the role of intra-treatment DCE MRI to assess and predict response to CRT. This may be related to the technical difficulties, e.g. tumor motion due to swallowing and workload required to process DCE data. We are aware of only two pilot studies which assessed changes in DCE parameters during radical radiotherapy in patients with HNSCC. Cao et al. reported an increase in PT blood volume (BV) 2 weeks into CRT to be associated with local control [21]. Baer et al. subsequently investigated a novel method of using parametric response maps of DCE MRI to predict survival following CRT in 10 patients: they found patients with a large percentage of PT gross volume that decreased in K^trans^ after 2 weeks were more likely to have significantly reduced survival [22]. Our larger study supported their findings that intra-treatment changes in K^trans^ is a potential biomarker in predicting treatment response.

In this study, we also investigated the role of SW-derived R_2_* as a predictive biomarker in HNSCC. SW MRI is an alternative, hypoxia-dependent, non-invasive imaging technique that exploits the paramagnetic properties of deoxyhaemoglobin in erythrocytes to create contrast. Our interest in SW MRI stems from pre-clinical data [23] and a previous clinical study in cervical cancer demonstrating the ability of baseline tumor R_2_* to predict response to CRT: responders had a lower average baseline R_2_* than non-responders [24]. This result was not replicated in our study and we did not find any apparent trends for alterations in R_2_* during the first 2 weeks of CRT. The only other R_2_* study in HNSCC was recently published by Min et al. and they also failed to demonstrate any clear pattern in its weekly changes throughout radiotherapy [25]. They did not correlate R_2_* with treatment outcome, but as with our observation, it was evident that R_2_* does not appear to have a predictive role in HNSCC as a standalone parameter. A possible explanation is that R_2_* values are strongly dependent on tumor BV [26, 27], which is highly heterogeneous in HNSCC. An accurate and robust measurement of tumor BV is challenging [28, 29]. Therefore, additional work is required to ascertain how best to interpret R_2_* measurements with BV before it can be utilized as a hypoxia imaging biomarker in HNSCC.

Attempts to combine multiple identified FMI parameters failed to yield superior predictive power over a single parameter (∆ADC at week 2) in this cohort. This may partly be due to the relatively small number of non-responders in our study (8/35, 23%). The risk of treatment failure is not truly binarised by a single parameter threshold, and in ‘real-life’ clinical practice, intra-treatment changes in other predictive parameters (TLG_40%_, K^trans^ and V_e_) may prove useful in further determining the risk in equivocal cases. Our study has provided the basic framework for early intra-treatment assessment with FMI in locally advanced HNSCC, but requires further refinement and validation with more patients. This work continues and we are expanding our PET and functional MRI database beyond the current study cohort.

There are limitations of this study. Eight patients with T1–2 tonsillar cancer did not have measurable PT following diagnostic tonsillectomy and it is unclear whether this would have an impact on the result. In addition, the cranio-caudal coverage of our MRI protocol meant that in five patients, involved LNs outside the FOV were excluded. However, the largest LN for each patient was included, which is likely to have been representative of the dominant tumor biology. Another difficulty was the requirement to exclude obviously necrotic or cystic regions of the tumor, which was performed manually.

## Conclusion

Our study highlighted the importance of intra-treatment scanning time points when integrated into clinical practice due to its impact on prediction outcome. This study provides the framework of utilizing multimodality FMI early during CRT and could be used to inform the design of future risk-stratified adaptive interventional studies in HNSCC.

## Electronic supplementary material

ESM 1 (DOCX 168 kb)
