# Supplementary material for: Changes in multimodality functional imaging parameters early during chemoradiation predict treatment response in patients with locally advanced head and neck cancer
Source: Eur J Nucl Med Mol Imaging. 2017 Nov 21;45(5):759–67. doi: 10.1007/s00259-017-3890-2 (PMC5978912; doi:10.1007/s00259-017-3890-2)
Supplement: Supplementary file 1 — (DOCX 168 kb) [file 259_2017_3890_MOESM1_ESM.docx]

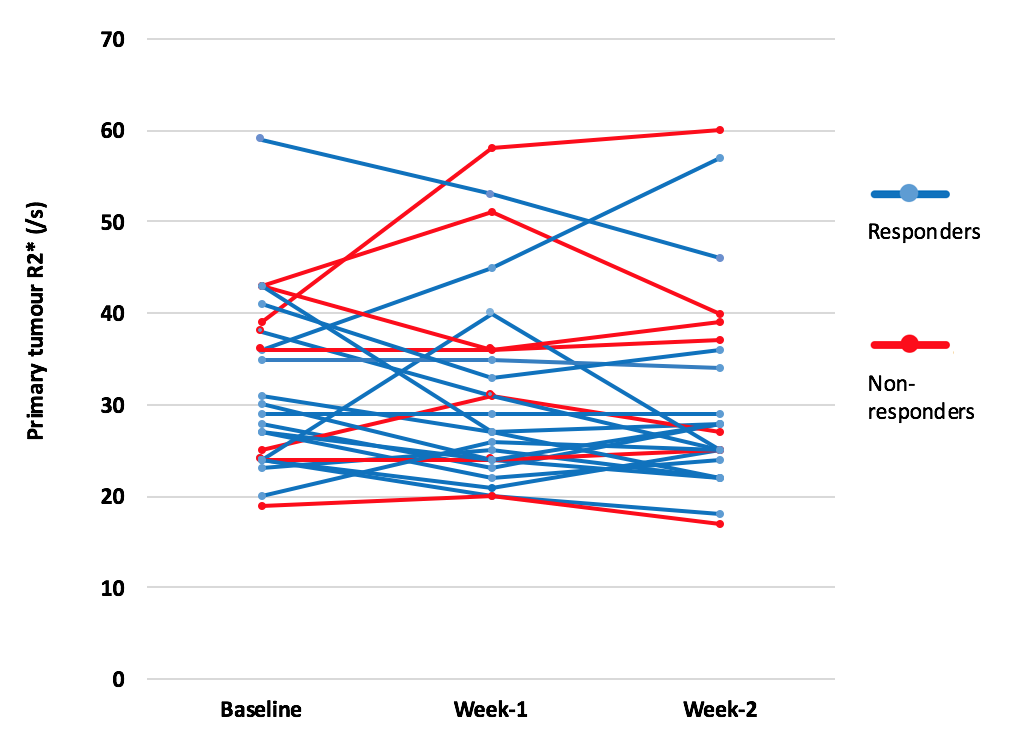


**Supplementary Figure 1.** Longitudinal changes in primary tumour R_2_* values early during radical chemoradiotherapy.

| **Supplementary Table 1. Comparison of clinical characteristics between responders and non-responders (n=35)** | | | |
| --- | --- | --- | --- |
| **Anatomical MR volume** | | | |
| **Combined** |  |  |  |
| Pre-treatment (cm^3^) | 17.3±11.6 | 30.2±19.6 | 0.174 |
| Δ post-wk1 | -27.0±23.4% | -15.0±21.9% | 0.278 |
| Δ post-wk2 | -37.4±25.9% | -28.0±30.7% | 0.564 |
| **Primary tumour** |  |  |  |
| Pre-treatment (cm^3^) | 11.2±8.9 | 24.7±15.8 | 0.062 |
| Δ post-wk1 | -32.8±24.3% | -8.9±17.8% | 0.139 |
| Δ post-wk2 | -56.8±24.9% | -21.4±30.3% | 0.174 |
| **Lymph nodes** |  |  |  |
| Pre-treatment (cm^3^) | 11.7±7.2 | 13.7±8.8 | 0.505 |
| Δ post-wk1 | -23.3±28.2% | -4.6±44.2% | 0.434 |
| Δ post-wk2 | -24.6±38.2% | -20.1±46.1% | 1.000 |

| **Supplementary Table 2. Comparison of clinical characteristics between responders and non-responders (n=35)** | | | |
| --- | --- | --- | --- |
| **Clinical features** | **Responders** | **Non-responders** | **P value** |
| **HPV** |  |  |  |
| Positive | 19 | 3 | 0.116 |
| Negative | 8 | 5 |  |
| **T classification** |  |  |  |
| T1-2 | 18 | 2 | 0.051 |
| T3-4 | 9 | 6 |  |
| **N classification** |  |  |  |
| N0-1 | 8 | 4 | 0.402 |
| N2-3 | 19 | 4 |  |
